# On the Occurrence and Morphology of Spontaneous Tumours in the Pituitary, Adrenals, Gonads and Mammary Glands in Rats Before and After Gonadectomy, Irradiation and Treatment with Oestrogen or Progesterone

**DOI:** 10.1038/bjc.1959.55

**Published:** 1959-09

**Authors:** Stig Kullander

## Abstract

**Images:**


### ON THE OCCURRENCE AND MORPHOLOGY OF SPONTANEOUS TUMOURS IN THE PITUITARY, ADRENALS, GONADS AND MAMMARY GLANDS IN RATS BEFORE AND AFTER GONADECTOMY, IRRADIATION AND TREATMENT WITH OESTROGEN OR PROGESTERONE

STIG KULLANDER

*From the Gynaecological Department, University Hospital, Lund, Sweden*

Received for publication June 26, 1959

MUCH suggests that hormones play a complex role in the pathogenesis of mammary tumours. In a closed colony of rats kept at the Gynaecological Department, University Hospital, Lund, tumours often occur spontaneously in the mammae of the older animals. It was therefore decided to study the animals systematically for tumours in endocrine organs (pituitary, adrenals and gonads), which might have some relation to mammary tumours.

Since castration and treatment with oestrogen or progesterone are known to affect the development of mammary tumours in rats (Geschickter, 1947 ; Burrows and Horning, 1952), the influence of such measures was also studied.

Roentgen irradiation, which has a carcinogenic effect, was also included in the investigation.

### MATERIAL AND METHODS

Virginal female rats and male rats that had since birth been kept separate from females, were observed, generally until their natural death (occasionally some of the animals were killed to provide material for histological examination). All the animals were raised alike in cages of the same size and on a diet of grain, milk and kitchen refuse.

Some male and female animals were gonadectomised at 3 weeks of age.

Two groups of spayed females received injections of either oestrogen (1·25 mg. oestradiolbenzoate in 0·25 c.c. oil) or progesterone (12·5 mg. progesterone in 0·25 c.c. oil) once a week from the age of 1 to 12 months.

A single X-ray dose over the whole body was given to some animals—intact and spayed females—when they were 3 weeks old. When combined with spaying the dose was given on the day after the operation. The " air dose " given was 300 r (filter 1 mm. Al, $\frac{1}{2}$ mm. Cu ; distance 50 cm., exposure time 3·9 minutes).

The animals were examined post mortem for any tumours. In none of the animals were any tumours found in the organs studied before the age of 12 months. The animals that lived for more than 12 months are listed below.

| | |
|---|---|
| Intact males | 49 |
| Intact females | 125 |
| Castrated males | 44 |
| Spayed females | 98 |
| Irradiated intact females | 67 |
| Irradiated spayed females | 45 |
| Oestrogen treated spayed females | 48 |
| Progesterone treated spayed females | 48 |
| Total | 574 |

Tumours were fixed in 10 per cent formalin and embedded in paraffin. The microscopical sections were stained with haematoxylin-eosin.

FIG. 1.—Hypophyseal tumours in different groups of rats. Each filled square indicates one rat dying from such a tumour. M and A designate co-existing tumours in the mammary glands and adrenals. The numerals give survivals in various age classes.

FIG. 2.—Adrenal tumours in different groups of rats. Each filled square indicates one rat dying from such a tumour. H and M indicate co-existing tumours in hypophysis and mammary glands. The numerals give the survivals in the various age classes.

### RESULTS

*Tumour frequency*

The incidence of tumours in the pituitary, adrenals and mammae in the various groups is given graphically in Fig. 1, 2 and 3.

In all the groups the frequency increased markedly with the ages of the animals.

In the statistical analysis the expected tumour development was assessed by placing the tumour frequency in relation to the number of " risk months " (the number of animals surviving a number of months). These expected values were compared with the number observed.

FIG. 3.—Mammary tumours in different groups of rats. Each filled square indicates one rat dying from such a tumour. H and A designate co-existing tumours in hypophysis and adrenals. The numerals give the survivals in the various age classes.

The expected and observed frequencies of tumour in spayed female rats with or without treatment with X-rays or progesterone—compared with intact females, intact and irradiated or spayed and oestrogen-treated—are given below :

| | Pituitary tumour | Adrenal tumour | Mammary tumour |
|---|---|---|---|
| Number of observed occurrences | 23 | 14 | 14 |
| Calculated No. of occurrences | 28·8 | 12·8 | 26·6 |
| Difference . . . . | −5·8 | + 1·2 | −12·6 |

The difference between the observed and expected values for mammary tumours was more than three times the mean error. The same tendency was found for the pituitary tumours, but the difference was only about 1·5 times the mean error. The difference in frequency of mammary tumours showed the same trend in all the months and in all of the experimental groups.

For the pituitary tumours the negative difference was statistically significant only for the progesterone-treated spayed female rats during the observation period of 18–26 months compared with the group treated with oestrogen.

Irradiation produced a statistically significant increase in the incidence of pituitary and mammary tumours, but not of adrenal tumours in the combined groups of intact-spayed females compared with irradiated intact-spayed females.

Intact and spayed females, intact irradiated and spayed irradiated females, and oestrogen-treated and progesterone-treated spayed females were compared in the same way for mammary tumour frequency. The observed tumour frequency in each group was compared with the expected frequency. An analysis of this kind shows a statistically significant increase of the number of tumours in intact compared with spayed females—with or without irradiation—and in oestrogen-treated spayed females against progesterone-treated. The opposite kind of difference, though not statistically certain was found on comparison of intact and castrated male animals.

A point of interest is that some tumours occur in combinations too often to be ascribed to chance. Thus 4 animals had had all 3 (pituitary, adrenal and mammary) kinds of tumours. This coincidence is remarkable.

The combination of pituitary-mammary tumours occurred in all the various types of animals studied and to a greater extent than was theoretically expected :

| | Observed number of animals with combined pituitary-mammary tumours | Expected number of animals with combined pituitary-mammary tumours |
|---|---|---|
| Intact and castrated males | 3 | 0·7 |
| Intact, spayed and spayed hormone-treated females | 5 | 3·4 |
| Irradiated females | 9 | 6·8 |

There was no such evidence of a concomitant, non-fortuitous coincidence of the combination adrenal tumour and mammary tumour.

On the other hand, the combination of pituitary and adrenal tumours revealed a preponderance that is also recorded in all the groups :

| | Observed number of animals with combined pituitary-adrenal tumours | Expected number of animals with combined pituitary-adrenal tumours |
|---|---|---|
| Intact and castrated males | 2 | 1·6 |
| Intact, spayed and spayed hormone-treated females | 5 | 3·1 |
| Irradiated females | 4 | 1·0 |

### EXPLANATION OF PLATE

FIG. 4.—Mammary tumour (fibroadenoma) in a female rat. H and E.
FIG. 5.—Mammary tumour (sarcoma in a female rat. H and E.
FIG. 6.—Mammary tumour (adenoma) in an irradiated intact female rat. H and E.
FIG. 7.—Mammary tumour in an irradiated spayed female rat. H. and E.

4

5

6

7

Ovarian tumours were observed in 4 intact, non-irradiated females at the age of 2 years. These animals had no tumours in the pituitary, adrenals or mammae. A testicle tumour was seen in an intact male rat, aged 38 months. Moreover, one also occurred in a testis remnant in a " castrated " male which had reached the age of 36 months (all other gonadectomized animals—males and females—had no visible gonadal vestiges when examined post mortem).

*Tumour morphology*

All the *pituitary tumours* observed were bluish-red and richly vascularized. They varied in size : some were as large as a hazel-nut, while others were much smaller or even minute and were then varified microscopically. The tumours seemed to press the cerebral tissue aside without infiltrating it. Animals with pituitary tumours were either markedly restless and irritable or somnolent. Balance disturbances were observed. Microscopically the pituitary adenomata were composed almost entirely of large, pale, chromophobe cells with large, pale nuclei and usually very distinct nucleoli. Both basophilic and—more rarely—eosinophilic cells were sometimes present. Congestion of the capillaries, larger vessels and sinusoids was common.

The *adrenal tumours* were all cortical, protruding from the surface or so large that the normal morphology of the gland was no longer discernible. In such cases the gland seemed to be completely buried in the tumours. No metastases were observed. The tumours were smooth surfaced and circumscribed. The cut surface of the tumours was mottled yellowish-red with massive yellow areas and large blood-filled sinusoids. Microscopically the tumours consisted of cells which, in some sections, were relatively small with a moderate amount of cytoplasm and small, dark, usually round nuclei, and in other sections large with pale, fatty cytoplasm and somewhat larger and paler nuclei. In some cases the cells were arranged in trabeculae or garland-like, sometimes in tubule-like patterns. Stroma was sparse. Practically all of the adrenocortical tumours had cavities filled with a faintly stainable mucinous fluid and resembling those sometimes seen in the testicular and ovarian tumours of these animals. The tumours also contained capillaries and larger vessels as well as blood-filled sinusoidal cavities. The structure of the cortical tumours in the adrenals did not vary with the types of animals studied.

The *testicular tumour* which had growth in a testicle vestige of an incompletely castrated rat was the size of a lentil, rounded and mottled, yellowish-red cut surface. In the intact male rat with a gonad tumour the distal part of one testicle had been completely involved by a tumour which both macroscopically and microscopically was of the same character as the vestigial-testicular tumour. The testicle on the other side was small. Its tubules were atrophic. Microscopically the tumours contained cells and tissue structures resembling those observed in granulosa cell-androblastoma type tumours in the ovaries (see below) and those seen in the cortical tumours of the adrenals. Relatively small cells with rounded, dark nuclei and, in some sections, fatty, large and pale cells were arranged either irregularly or in trabeculae, sometimes tubule-like. Large sinusoidal cavities filled with blood or faintly stainable fluid were common. Four *ovarian tumours* were observed in intact female animals, but no tumours were found in those that were irradiated. They were all of granulosa cell-androblastoma type (Kullander, 1956).

The *mammary tumours* observed were all well circumscribed and mobile. No metastases were seen. They ranged in size from that of a hazel-nut to that of a goose's egg. In some instances the skin over the tumour ulcerated owing to its being rubbed against the floor or walls of the cage.

The microscopic structure was the same in eight out of nine tumours observed in intact females : fibroadenoma with rich development of glandular parenchyma and with histological signs of secretion (Fig. 4). A milky fluid oozed from the cut surface of the tumours. One mammary tumour had the character of a sarcoma (Fig. 5).

Only one tumour was observed in spayed females. There was extremely little glandular parenchyma and the microscopic picture closely resembled that of a fibroma.

There were no mammary tumours among intact males. Two of the 4 mammary tumours observed in castrated males—3 cases in combination with pituitary tumours—showed the same microscopical picture as the fibroadenomata in intact females, whereas the other 2 resembled the fibroma in spayed females.

Some mammary tumours preponderantly of sarcoma type were seen in the oestrogen-treated spayed females. The cells were fusiform or rounded with pale, relatively abundant cytoplasm and large nuclei. Mitoses were common. Other tumours were fibroadenomata with signs of secretion.

Irradiated intact females had mostly fibroadenomata, histologically resembling those found in untreated, intact females. One mammary tumour was of sarcoma type. Some tumours resembled adenomata (Fig. 6).

Irradiated spayed females had several mammary tumours with solid groups of epithelial tumour cells in a scanty stroma, showing hardly any signs of secretion (Fig. 7).

### DISCUSSION

Reports of pituitary tumours—chromophobe adenomata—in old rats are on record (Wolfe, Bryan and Wright, 1938 ; Saxton, 1941 ; Saxton and Graham, 1944). In the Yale strain a pituitary adenoma was hardly ever seen in rats younger than 400 days. After this age the frequency increased with age to about 60 per cent for male rats and 30 per cent for females older than 600 days (Saxton and Graham, 1944). In the present study the youngest animal in which a tumour was observed was 12 months old (Fig. 1). Pituitary tumours were equally common in both sexes. No certain difference was found between gonadectomised and intact animals—either males or females—in respect to the occurrence of pituitary tumours. Cessation of production of thyroid hormone (surgical or radiological thyroidectomy) may increase the incidence of pituitary tumours of chromophobe character in mice, probably with almost exclusive secretion of thyrotropin (Furth *et al.*, 1955). Some chromophobe pituitary adenomata in mice have been presumed to produce mainly ACTH. These tumours, growing after irradiation of the whole body, are thought to be the indirect result of damaged adrenals with reduced cortical hormone production (Furth, Gadsen and Upton, 1953). Ceased gonad hormone production in the rats in the present material did not seem to favour the development of pituitary tumours. The growth of pituitary tumours in female rats, both intact and spayed, however, was promoted by total body irradiation. Previously this has been observed only in intact mice (Upton and Furth, 1953, 1955).

Injection of oestrogens into rats is known to increase the frequency of pituitary adenomata (Burrows and Horning, 1952). In the present material oestradiol treatment of spayed rats induced more pituitary adenomata than progesterone-treatment. The injections of oestradiol, which was stopped 7 months before the first pituitary tumour appeared in this group of animals, seems to have started irreversible pre-tumorous changes in the pituitary at an early stage.

In the rats investigated the pituitary tumours appeared to develop somewhat earlier than the mammary tumours, besides which there was a statistically significant correlation between pituitary and mammary tumours. Morphological-functional disorders of the pituitary thus appear to affect the mammary glands and favour the growth of tumours in them. The existence of such a connection is also suggested by other observations. Mice with chromophobe pituitary adenomata acquire alveolar hyperplasia and distension of the mammary gland ducts, a change which can also be seen though less marked, in elderly female mice without pituitary tumours (Upton and Furth, 1955). In rats of the Albany strain mammary fibroadenomata are fairly common, but not in the Vanderbilt strain. The anterior lobe of the pituitary in the Albany strain contains a much larger number of chromophobe cells. This is also a characteristic of elderly animals. Albany animals thus present signs of accelerated ageing in the pituitary (Wolfe and Wright, 1943).

Adrenal tumours—usually with a later age maximum than the pituitary adenomata—also occurred in a definite connection with pituitary tumours in the rats in the present investigation and may thus be secondary to a primary pituitary disorder. Although all pituitary adenomata seemed to have a similar morphology with principally chromophobe cells, it is possible that they had a varying production of hormones : mainly prolactin when combined with mammary adenomata — predilectively ACTH and/or gonadotrophins when combined with adrenal tumours.

Gonadectomy increases the frequency of cortical tumours in the adrenals in both male and female rats (Houssay *et al.*, 1951). This could not be demonstrated with certainty in the present material. Tumours in the adrenal cortex of rats produce oestrogens, progesterone and androgens (Houssay, 1954). Among the spayed females with adrenal tumours there were indications of stimulation of the uterus. The production of hormones in these tumours, however, is not of such a quality or quantity as to stimulate the development of mammary tumours. There was no trace of a connection between adrenal and mammary tumours in any of the groups of this material.

Total body irradiation was not seen to increase the frequency of adrenal tumours in the females.

In this investigation neither oestradiol nor progesterone proved to have any influence on the occurrence of adrenal tumours. Ambrad-Dominguiez and Cardez (1952) state that administration of oestradiol to spayed rats inhibits the occurrence of adrenal tumours, whereas progesterone and deoxycorticosteron are ineffective.

Spontaneous, endocrinously active ovarian tumours in rats are rare, but can be induced experimentally by implantation of ovarian tissue into the spleen of gonadectomized animals (Kullander, 1956). Roentgen irradiation of intact female rats caused no tumours in the ovaries, nor were Drips and Ford (1932) able to induce ovarian tumours in rats by X-ray.

Leydig-cell tumours have been observed after implantation of testicle tissue in the spleen of castrated animals (Twombley, Meisel and Stout, 1949). Biskind and Biskind (1945), who also implanted testicular tissue in the spleen of castrates observed tumour growth in one. Its histological structure resembled what was observed in the 2 cases of spontaneous testicular tumour in the present investigation, i.e. granulosa cell-androblastoma tumour.

The mechanism of tumour induction in animals in which ovarian or testicular tissue had been implanted into the spleen at gonadectomy is assumed to be due to an increased gonadotrophic stimulation after the cessation of influence of pituitary-antagonistic gonadal hormones. These hormones are largely inactivated in the liver. It is possible that a considerable reduction of the gonad parenchyma may lead to a similar hormonal imbalance, which would explain the appearance of tumour in the testicular fragment of the incompletely castrated rat.

It was noteworthy that morphologically—and possibly functionally—similar tumours occur spontaneously in these rats in the testicles and ovaries as well as in the adrenal cortex. This might suggest that similar cells—capable of forming tumours induced by pituitary stimulation—are to be found in the gonads and the adrenal cortex.

Administration of oestradiol to spayed females caused an increased number of mammary tumours, thus verifying earlier observations (Geschickter, 1947). Observations on transplanted mammary fibroadenomata in rats (Millar and Noble, 1954) have shown that such transplants grow more quickly in female hosts. If the male or female host is gonadectomized, the growth of the adenoma-transplant is increased or decreased, respectively. In accordance with earlier observations (Heiman, 1943) the effects of progesterone are not such as to increase the number of mammary tumours in rats.

Total body irradiation of both intact and spayed female rats resulted in a large number of mammary tumours. But Furth and Furth (1936) and Furth and Butterworth (1936), who irradiated mice at the age of 5–12 weeks with 200–400 r over the whole body, observed a falling frequency of mammary tumours. Passoneau, Brues and Hamilton (1953), however, have reported increased occurrence of fibroadenomata in the mammary glands of rats exposed to local skin irradiation.

### SUMMARY

Analysis was made of the frequency and morphology of spontaneous tumours in the pituitary, adrenals, gonads and mammary glands of a closed colony of rats. Besides intact females and males the material included animals gonadectomized at the age of three weeks, intact and spayed females exposed to roentgen irradiation of the entire body at three weeks of age, as well as spayed females treated with oestrogen or progesterone from the age of one to twelve months.

1. Spaying was followed by a reduction in the occurrence of mammary tumours. Mammary tumours were observed in castrated but not in intact males.

2. Oestrogen-treated spayed females acquired a larger number of pituitary tumours than progesterone-treated.

3. Irradiation increased the occurrence of pituitary and mammary tumours in both intact and spayed females. Adrenal tumours did not increase in frequency, nor were any ovarian tumours observed after irradiation.

4. The microscopical picture of 2 testicular tumours resembled that of some granulosa cell-androblastoma tumours found in the ovaries and of a number of tumours of the adrenal cortex.

5. The combination pituitary-mammary tumours and pituitary-adrenal tumours occurred among the various types of animals to an extent that cannot be explained as fortuitous coincidences.

Morphological-functional disorders in the pituitary in association with tumours in the adrenals, gonads and mammary glands in rats are discussed.

I thank Prof. C. E. Quensel, Lund, for help with the statistical treatment of the data.

### REFERENCES

AMBRAD-DOMINGUEZ, N. AND CARDEZA, A. F.—(1952) *Rev. Soc. argent. Biol.*, **28**, 129.
BISKIND, M. S. AND BISKIND, G. R.—(1945) *Proc. Soc. exp. Biol. N.Y.*, **59**, 4.
BURROWS, H. AND HORNING, E. S.—(1952) Oestrogens and Neoplasia. Oxford (Blackwell).
DRIPS, D. G. AND FORD, F. A.—(1932) *Surg. Gynec. Obstet.*, **55**, 596.
FURTH, J. AND BUTTERWORTH, J. S.—(1936) *Amer. J. Cancer*, **28**, 66.
*Idem*, DENT, J. N., BURNETT, W. T. AND GADSEN, E. L.—(1955) *J. clin. Endocrin.*, **15**, 81.
*Idem* AND FURTH, O. B.—(1936) *Amer. J. Cancer*, **28**, 54.
*Idem*, GADSEN, E. L. AND UPTON, A. C.—(1953) *Proc. Soc. exp. Biol. N.Y.*, **84**, 253.
GESCHICKTER, Ch. F.—(1947) ' Diseases of the breast.' Baltimore. (Lippincott).
HEIMAN, J.—(1943) *Cancer Res.*, **3**, 65.
HOUSSAY, B. A.—(1954) *Congr. period int. Gynéc. Obstet.*
*Idem*, B. A., CARDEZA, A. F., PINTO, R. M. and BURGOS, M. H.—(1951) *C.R. Soc. Biol., Paris*, **145**, 1712.
KULLANDER, S.—(1956) *Diss. Univ. Lund.*
MILLAR, M. J. and NOBLE, R. L.—(1954) *Brit. J. Cancer*, **8**, 495.
PASSONEAU, J. V., BRUES, A. M. AND HAMILTON, K. A.—(1953) *Proc. Amer. Ass. Cancer Res.*, **1**, 41.
SAXTON, J. A.—(1941) *Cancer Res.*, **1**, 277.
*Idem* AND GRAHAM, J. B.—(1944) *Ibid.*, **4**, 168.
TWOMBLEY, G. H., MEISEL, D. AND STOUT, A. P.—(1949) *Cancer*, **2**, 884.
UPTON, A. C. AND FURTH, J. (1953) *Proc. Soc. exp. Biol. N.Y.*, **84**, 255.—(1955) *J. nat. Cancer Inst.*, **15**, 1005.
WOLFE, J. M. AND WRIGHT, A. W.—(1943) *Cancer Res.*, **3**, 497.
*Idem*, BRYAN, W. R. AND WRIGHT, A. W.—(1938) *Amer. J. Cancer*, **34**, 352.


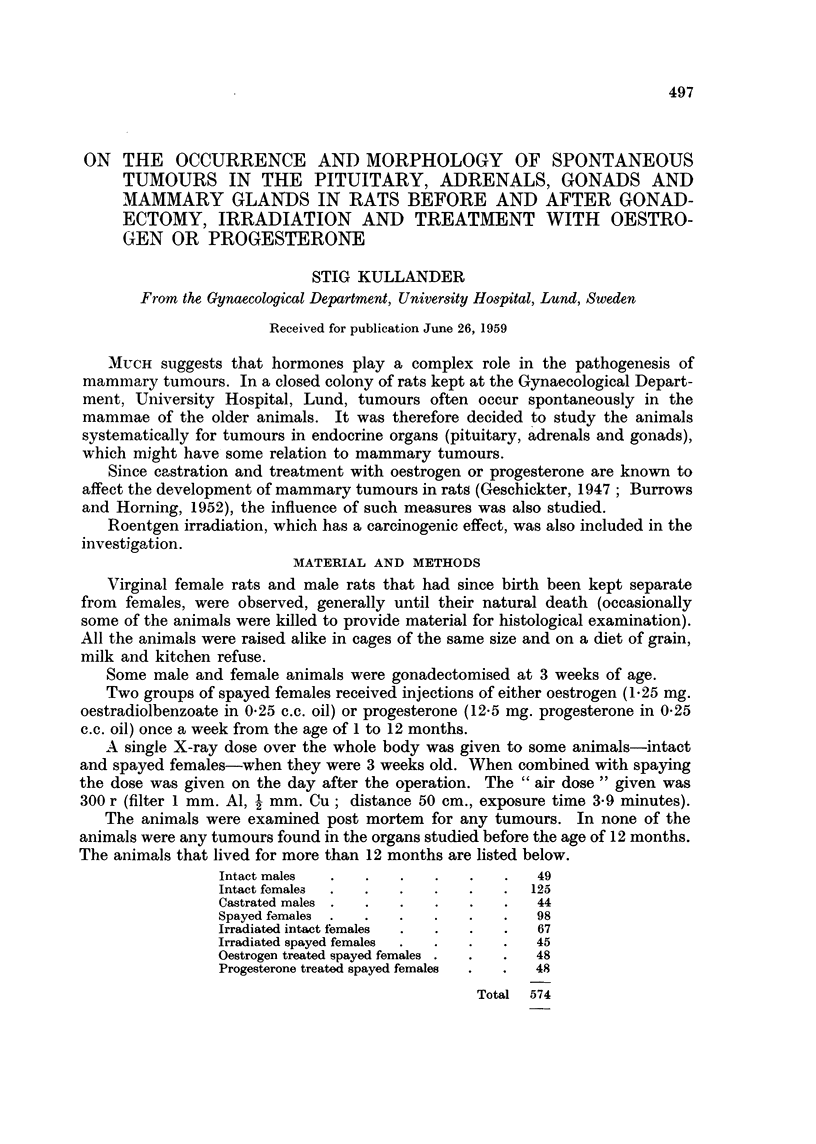

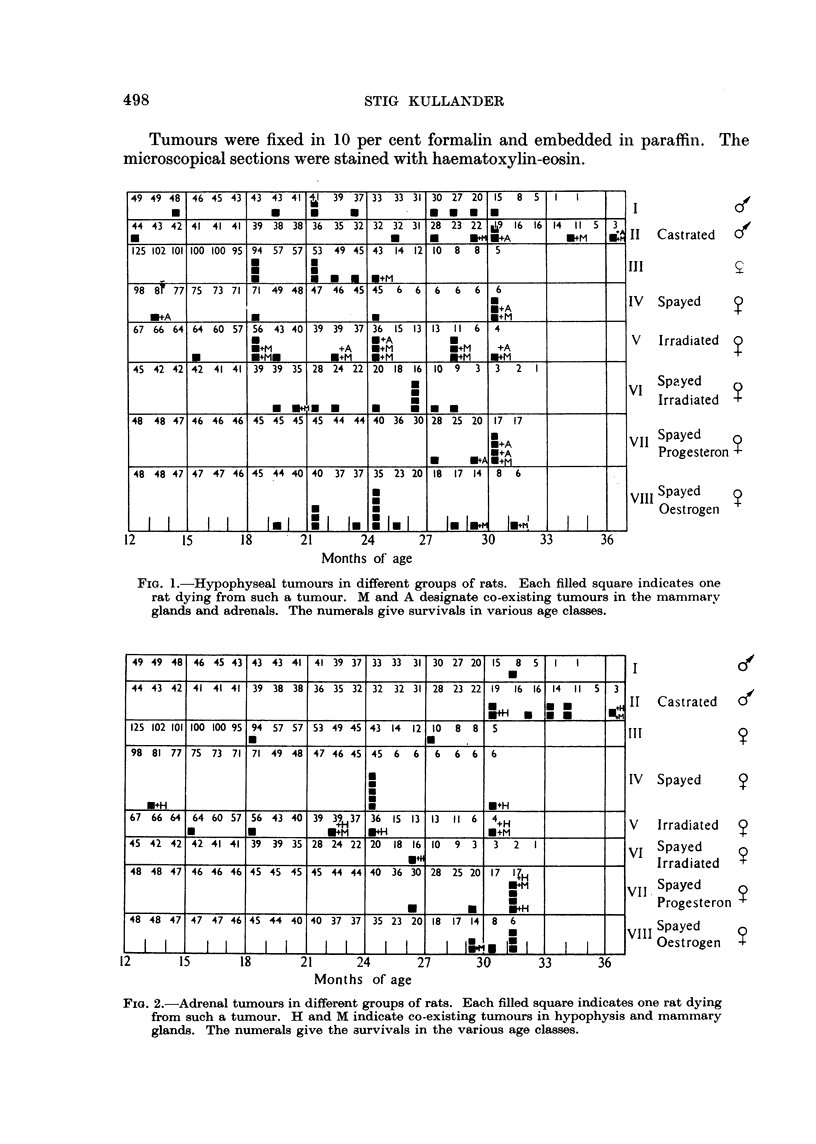

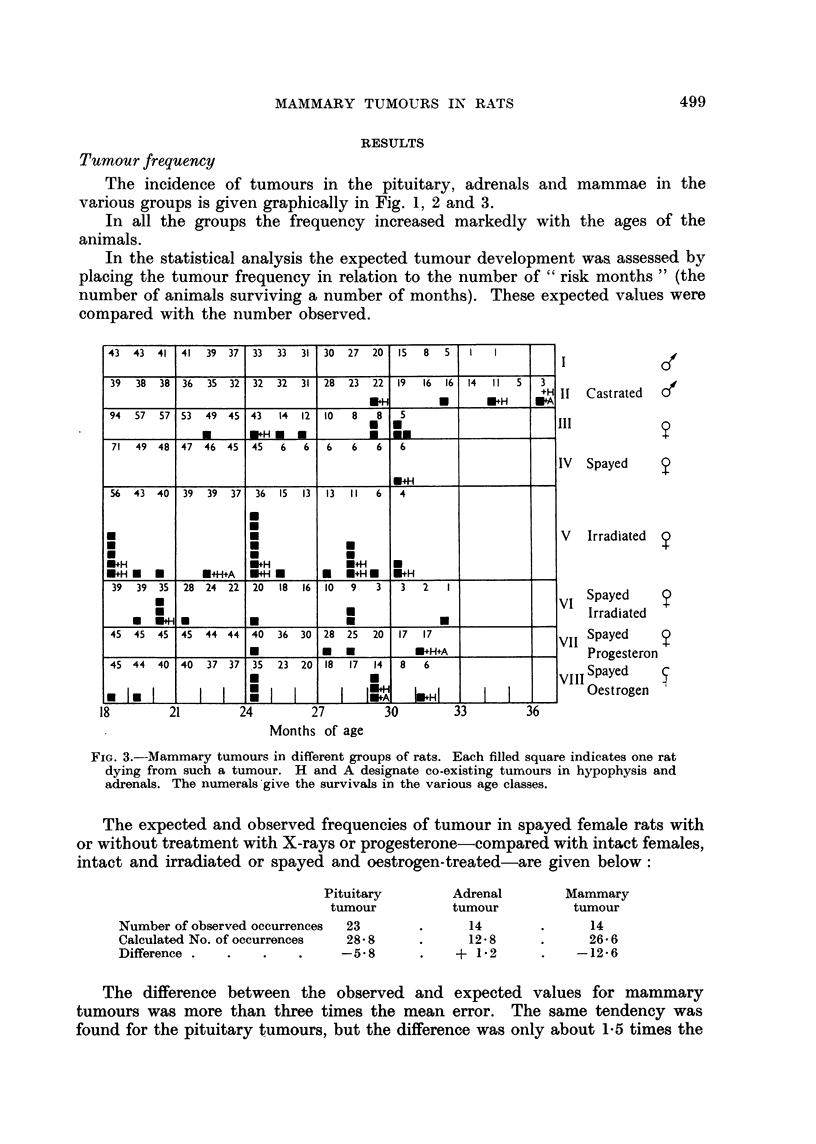

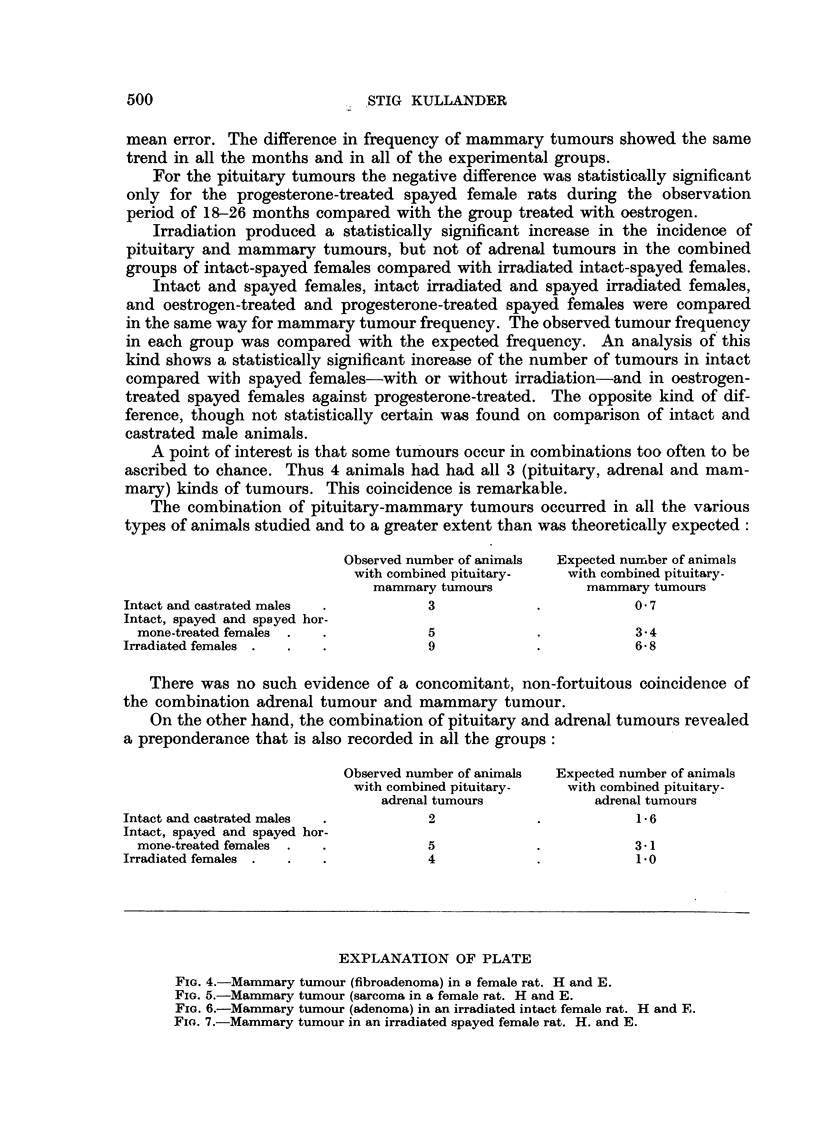

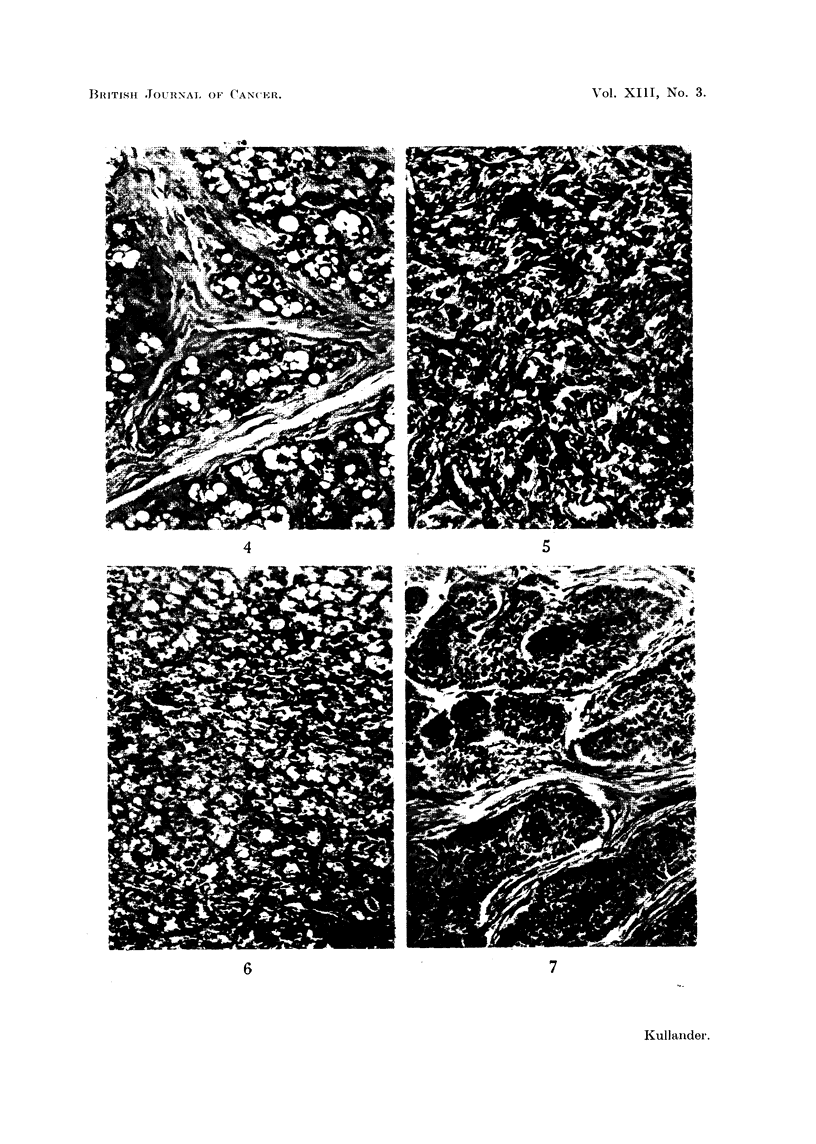

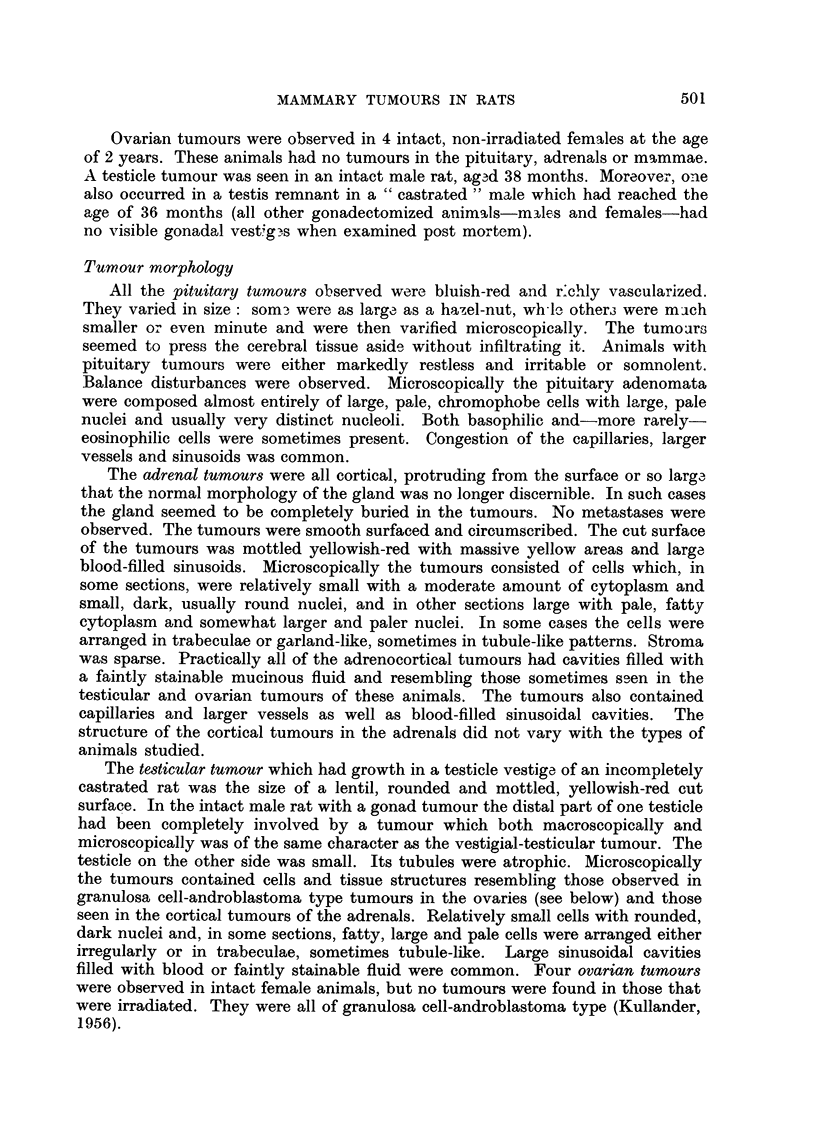

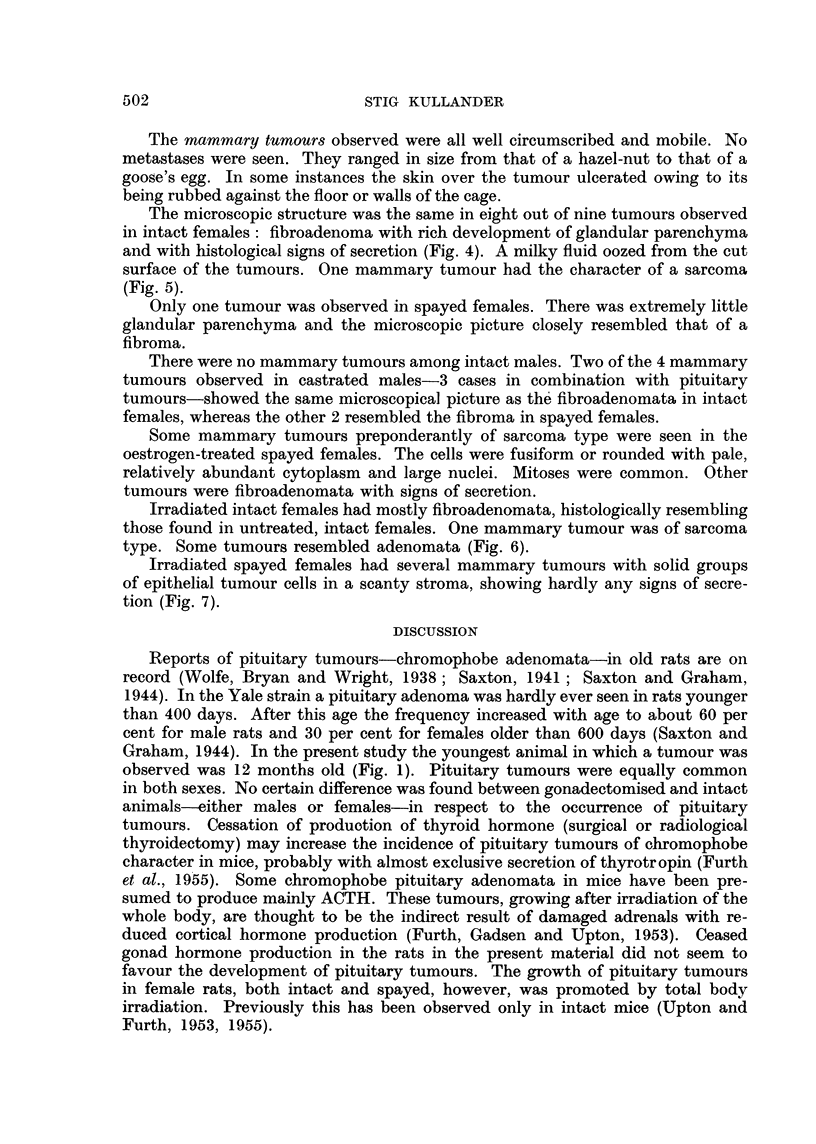

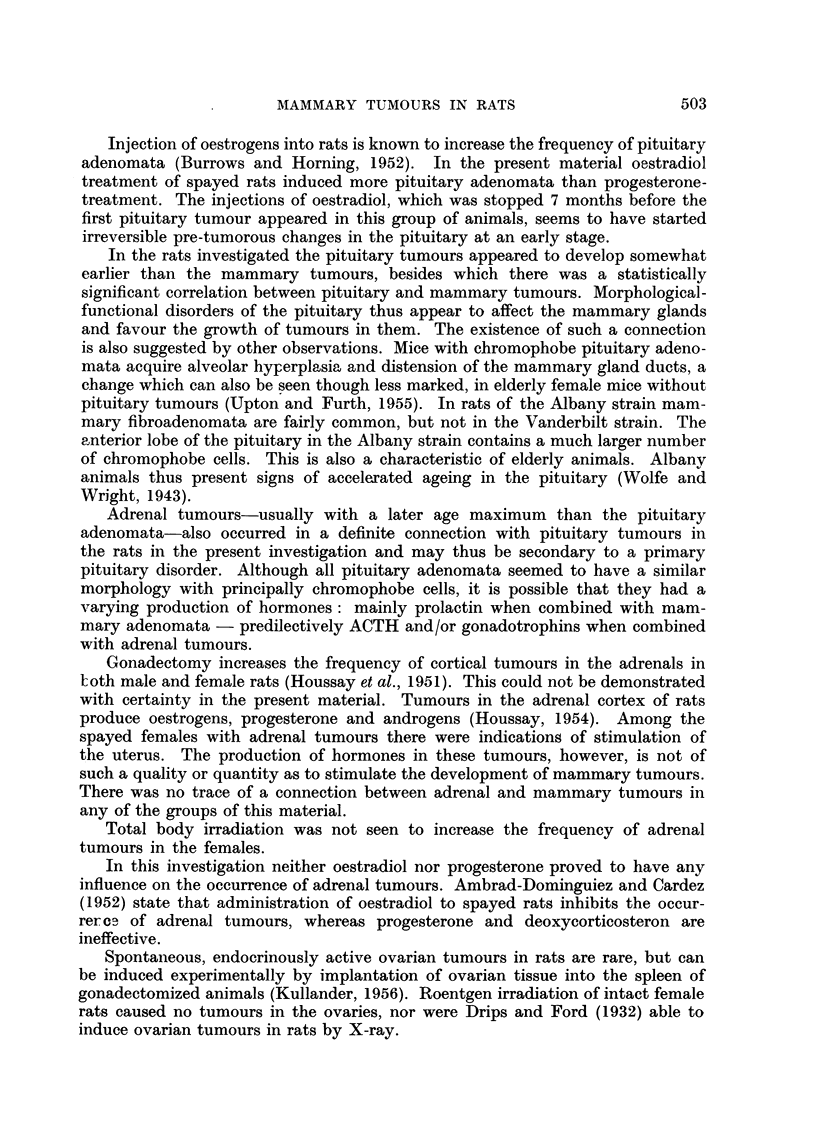

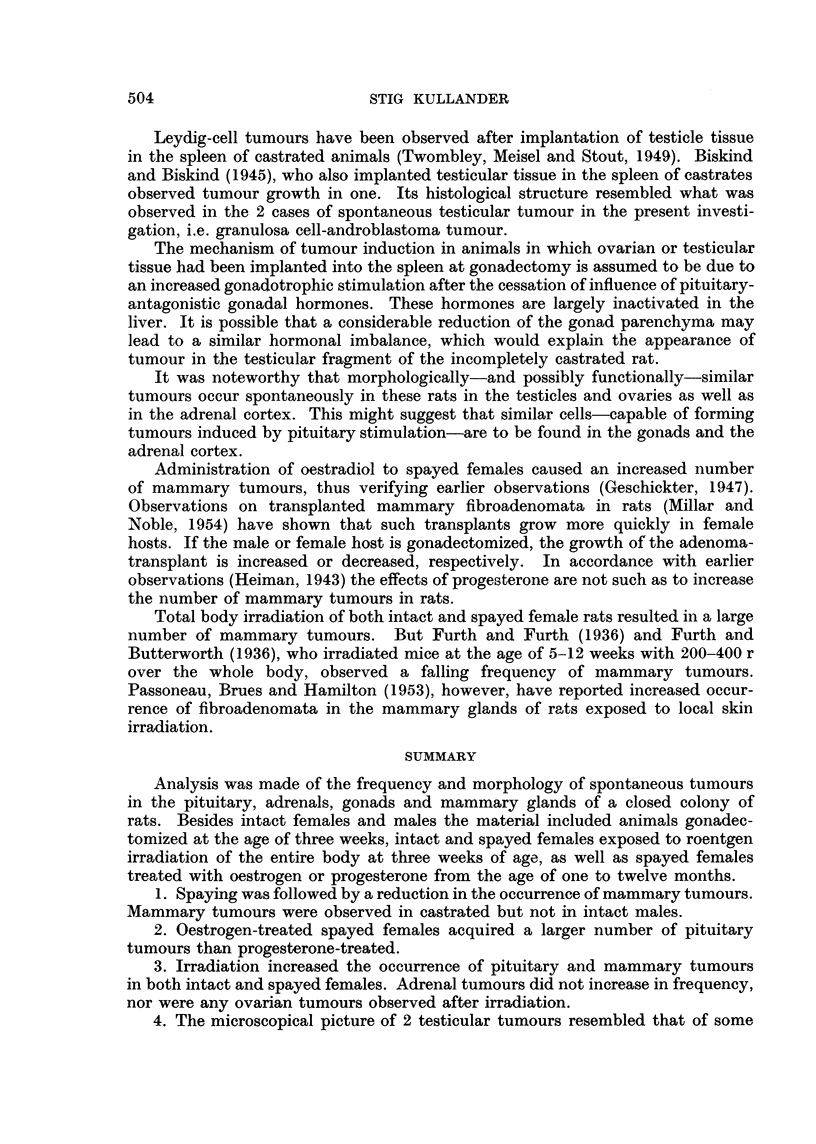

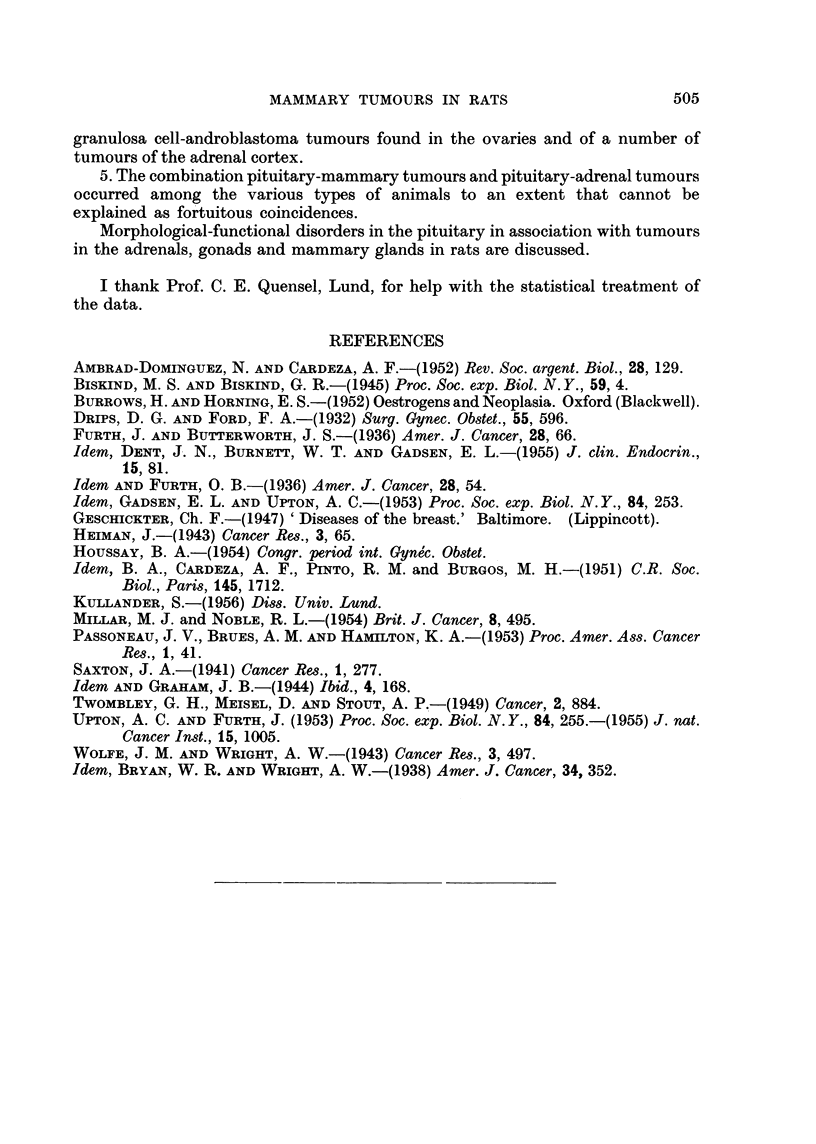


## References

[OCR_00668] AMBRAD DOMINGUEZ N., CARDEZA A. F. (1952). Acción del estradiol y desoxicorticosterona sobre los tumores suprarrenales de las ratas castradas.. Rev Soc Argent Biol.

[OCR_00681] FURTH J., GADSEN E. L., UPTON A. C. (1953). ACTH secreting transplantable pituitary tumors.. Proc Soc Exp Biol Med.

[OCR_00687] HOUSSAY B. A., CARDEZA A. F., PINTO R. M., BURGOS M. H. (1951). Tumeurs surrénales et actions oestrogéniques chez les rats blancs castrés.. C R Seances Soc Biol Fil.

[OCR_00693] MILLAR M. J., NOBLE R. L. (1954). Effects of exogenous hormones on growth characteristics and morphology of transplanted mammary fibroadenoma of the rat.. Br J Cancer.

[OCR_00705] UPTON A. C., FURTH J. (1955). Spontaneous and radiation-induced pituitary adenomas of mice.. J Natl Cancer Inst.

